# Nourishing the evidence: exposing bias and filling gaps in isocaloric intermittent fasting research—An opinion

**DOI:** 10.3389/fnut.2025.1563017

**Published:** 2025-03-11

**Authors:** Mohammed Hamsho, Wijdan Shkorfu, Yazan Ranneh, Abdulmannan Fadel

**Affiliations:** ^1^Department of Nutrition and Dietetics, Faculty of Health Sciences, Istanbul Yeni Yüzyil University, Istanbul, Türkiye; ^2^Department of Nutrition and Dietetics, Faculty of Health Sciences, Bahçeşehir University, Istanbul, Türkiye; ^3^Department of Nutrition and Dietetics, College of Pharmacy, Al-Ain University, Abu Dhabi, United Arab Emirates; ^4^Department of Nutrition and Health, College of Medicine and Health Sciences, United Arab Emirates University, Al Ain, United Arab Emirates

**Keywords:** intermittent fasting, calorie restriction, isocaloric, methodology, blinding, selection bias, expectation bias, cross-over

## Introduction

Intermittent fasting (IF) has become a widely recognized dietary pattern with potential health benefits. While calorie restriction (CR) focuses on regulating the amount and nutritional quality of food consumed, IF primarily targets the timing of food intake. IF includes various approaches, such as alternate-day fasting and time-restricted eating. A variety of IF protocols offer flexibility for individuals to adopt fasting schedules that suit their needs. Furthermore, IF aligns with many traditional and religious fasting practices, such as Ramadan or Lent, which demonstrate cultural adaptability and historical relevance. Integrating IF within culturally appropriate dietary frameworks offers a sustainable approach to health management, promoting adherence while respecting individual and community traditions (1).

The potential health benefits of IF are still in the early stages of being established. While IF may offer not only an enhanced form of CR but also unique metabolic advantages, IF and CR may exert some of their benefits through distinct physiological mechanisms. However, the rise of various fasting fad diets in popular media has blurred the distinction between evidence-based research and unverified claims (2). Most existing research on fasting has been conducted on animal models, and the evidence supporting health improvements in humans remains preliminary. The increasing interest in IF within the research community parallels a notable rise in popular literature and media coverage, establishing it as a hotspot area of inquiry.

Recent studies have illuminated several benefits associated with IF, including improved weight management, enhanced cardiometabolic health, and potential longevity. A search of the PubMed database indicates that ~46,461 results related to IF have been published from 1822 to the present (October 2024). Furthermore, recent investigations reveal a steady increase in research output focused on IF, with the United States leading this field (3).

Given the growing number of studies and the rising interest in their findings, it is imperative to critically assess the design of these investigations before drawing conclusions or making recommendations to the public or health sector. In this opinion piece, we aim to distinguish possible biases inherent in isocaloric IF vs. CR research, particularly those related to study design, and propose solutions for future studies.

## Challenging biases in IF studies

Randomized controlled trials (RCTs) are considered the gold standard for evaluating dietary interventions. Randomization ensures that studied subjects have an equal chance of being assigned to any intervention group, thus reducing selection bias. In addition, blinding is a method that is used in RCTs by which studied subjects and researchers are unaware of the intervention assignments, minimizing expectation biases and improving the validity of results. However, in certain dietary studies including IF, blinding has unique challenges. While pharmaceutical trials can easily utilize a placebo for blinding, dietary interventions often require participants to have a knowledge of their assigned regimen (4, 5). For example, comparing high-fat and high-carbohydrate diets cannot be effectively blinded. Similarly, IF participants are likely aware of their fasting regimen, influenced by prior knowledge, social media, or cultural associations.

To address this challenge, scholars should consider blinding the hypothesis rather than the intervention. Concealing certain information regarding expected outcomes can effectively mitigate participants' behavioral and perceptual biases, thereby reducing the impact of expectation bias (6, 7). For instance, informing participants that high-fat and high-carbohydrate diets yield equivalent health benefits may discourage them from modifying other lifestyle habits.

## Expectation biases

An example of the impact of expectation bias is found in a study entitled “Studying a Possible Placebo Effect of an Imaginary Low-Calorie Diet,” which investigated the impact of psychological/behavioral components of a weight loss intervention. The study subjects were obese and underwent a similar exercise program. The intervention group was informed that they were under a hypocaloric diet with a 5,500 kcal deficit weekly. On the other hand, the control group was informed that they were on a maintenance diet. Both groups consumed the exact amount of calories, but the intervention group lost significantly more body weight. The study authors concluded that “the opportunity of being allowed to participate in an experiment, which is supervised and controlled by professional dietitians and strength-training coaches, could have been a great stimulus for some of the participants to reduce their calorie intake and/or energy expenditure further than prescribed and lose weight as a result (8).

Furthermore, expectation bias may have appeared in IF studies due to preconceived notions about fasting that affect participants in RCTs. These notions are shaped by social media, study investigators, or religious teachings. In addition, previous experience with interventions such as fasting could be another significant factor in increasing the expectancy of participants (9). This bias may lead to an overestimation of intervention effects such as unintentional caloric restriction or improved adherence to healthy behaviors. For example, a large study conducted by Liu et al. (10) and published in JAMA compared IF combined with CR and CR alone. Even though both groups were instructed to consume the same caloric intake, the IF group reported consuming 138 fewer calories per day. This might be explained by participants extending the fasting window for 2.5 h/day beyond the prescribed period (10).

Moreover, another study in JAMA conducted by Jamshed et al. (11) found no difference in total calorie intake according to the remote food photography method, despite the IF group losing an additional 2.3 kg. The authors have further applied *post-hoc* analysis to estimate actual changes in energy intake using Hall et al.'s equations for modeling weight loss over time, which is reported to be more accurate than estimating energy intake from food records. *Post-hoc* analysis revealed that the fasting group consumed 214 kcal less compared to the CR group, which also explains the difference in total weight loss between the groups. In addition, the authors mentioned that the IF group extended the fasting duration by 4.8 h (11). This highlights the potential influence of expectation bias, where participants may subconsciously alter their dietary attitudes in line with their beliefs about the intervention.

## Selection bias

Selection bias arises when participants recruited for a study do not accurately reflect the characteristics of the wider population. Selection bias has been of concern in IF research, where individuals with previous fasting experience or positive perceptions of IF are more likely to participate. Such self-selection could exaggerate the benefits of IF since these individuals possess inherently favorable metabolic profiles or exhibit higher levels of motivation. Moreover, inadequate allocation concealment during randomization can lead to selective enrollment based on prognostic factors, skewing the results. Allocation concealment was shown to be poor in IF studies that compared isocaloric IF and CR (12). In general, studies with unclear or inadequate allocation concealment have been shown to overestimate treatment effects by up to 41% (13).

## Proposed solutions to reduce biases

While the reliance on self-reported dietary assessments may introduce recall bias among participants, it remains the sole feasible method for evaluating dietary intake in RCTs. Implementing rigorous RCTs that meticulously regulate dietary intake, physical activity, and fasting windows is prohibitively expensive and often poorly adhered to by participants. As a result, the long-term effects of IF remain insufficiently characterized.

In light of these challenges, crossover trials offer a promising solution to mitigate both expectation and selection biases. A crossover study design allows participants to experience both interventions (for example: IF and CR) in separate phases with a washout period. For instance, one group could follow IF for 8 weeks, while another group adheres to CR, followed by a 2-week washout before switching interventions. By exposing participants to both conditions, the crossover design effectively eliminates the influence of self-selection and facilitates direct within-subject comparisons, thereby minimizing expectation bias. However, it is essential to note that the long-term effect of the first intervention may persist into the second phase, suggesting the need for careful management of the washout period (14). In addition to the biases we mentioned, stricter crossover studies that control calorie intakes, eating windows, physical activities, and sleep cycles can eliminate recall bias. To the best of our knowledge, only one study of this type, which potentially resulted from participants' food recall, was published recently, aiming to evaluate the potential mechanisms through which time-restricted feeding produces negative energy balance.

Although the study had several limitations in the context of long-term isocaloric IF in adults with chronic diseases, it provides novel mechanistic insights not observed in RCTs (15). Therefore, more studies carrying out similar concepts on different population subgroups are needed.

Researchers must prioritize transparent reporting of randomization and allocation concealment methods. Clear descriptions of participant selection and randomization processes are essential for assessing study quality. In addition, pre-registration of study protocols and adherence to reporting guidelines such as CONSORT can further improve the credibility of findings.

## Discussion

While IF is undoubtedly effective for reducing caloric intake and promoting weight loss, its perceived superiority over other dietary interventions warrants scrutiny. Expectation and selection biases, coupled with methodological limitations, obscure the true effects of IF. Addressing these biases is critical not only for advancing scientific understanding but also for ensuring that public health recommendations are grounded in robust evidence. Paradoxically, the same biases that undermine research validity may enhance real-world outcomes. For example, expectation bias could motivate individuals to adhere more strictly to an IF regimen, which may indirectly lead to improved health outcomes. However, this hypothesis requires further investigations to determine whether such effects are sustainable and beneficial in the long term. By adopting rigorous methodology and acknowledging potential biases, researchers can build a more reliable evidence base for IF. This will enable healthcare professionals to provide evidence-based recommendations, ultimately benefiting individuals seeking sustainable dietary strategies. In conclusion, the rapid increase in IF research underscores the need for critical appraisal of its methodological rigor. By addressing biases through innovative study designs and transparent reporting, the scientific community can better demonstrate the mechanisms and health benefits of IF. This will pave the way for more accurate, evidence-based dietary guidelines, bridging the gap between research and real-world application. Future research should emphasize incorporating randomized allocation concealment procedures with standardized outcome measures. This strategic focus will not only enhance the reliability of intermittent fasting studies but also contribute significantly to the broader discourse surrounding dietary interventions.
